# AAT score based on pretreatment indicators predicts outcomes in unresectable HCC patients treated with TACE, Sintilimab, and Bevacizumab

**DOI:** 10.3389/fonc.2026.1867932

**Published:** 2026-06-10

**Authors:** Wanying Qin, Xudong Fei, Renfang Shi, Yuchen Zhou, Jiajie Tang, Yizhe Liu, Yi Zhu, Liya Suo, Weijia Liao

**Affiliations:** 1 Laboratory of Hepatobiliary and Pancreatic Surgery, The First Affiliated Hospital of Guilin Medical University, Guilin, Guangxi, China; 2 Department of Clinical Nutrition, The First Affiliated Hospital of Guilin Medical University, Guilin, Guangxi, China; 3 Department of General Surgery, Integrated Hospital of Traditional Chinese Medicine, Southern Medical University, Guangzhou, Guangdong, China; 4 Department of Hepatobiliary Surgery, Nanfang Hospital, Southern Medical University, Guangzhou, Guangdong, China; 5 Department of Hepatobiliary Pancreatic Surgery, The First Affiliated Hospital of Guilin Medical University, Guilin, Guangxi, China; 6 State Key Laboratory of Targeting Oncology, National & Guangxi Key Laboratory of Bio-targeting Theranostics, Guangxi Medical University, Nanning, Guangxi, China

**Keywords:** Bevacizumab, prognostic risk model, Sintilimab, transcatheter arterial chemoembolization, unresectable hepatocellular carcinoma

## Abstract

**Background:**

Hepatocellular carcinoma (HCC) is the second leading cause of cancer-related death in China, characterized by insidious onset and poor prognosis. Most patients are diagnosed at intermediate-advanced stages with unresectable HCC (uHCC), and the combination of transarterial chemoembolization (TACE) with targeted and immunotherapy has shown promising efficacy. This study aimed to develop a prognostic tool for uHCC patients receiving TACE combined with sintilimab and bevacizumab therapy.

**Methods:**

A total of 176 uHCC patients from two institutions (March 2021 to May 2024) were included. Univariate and multivariate Cox regression identified alpha-fetoprotein (AFP), alkaline phosphatase (ALP), and tumor burden score (TBS) as independent predictors of overall survival (OS), based on which the AAT score model was constructed. The area under the receiver operating characteristic (ROC) curve in training and validation cohorts was calculated, and Kaplan-Meier analyses were performed to evaluate the model.

**Results:**

Cox regression confirmed AFP, ALP, and TBS as independent OS predictors, with the AAT model achieving areas under the ROC curve of 0.813 and 0.819 in training and validation cohorts, outperforming individual factors. Kaplan-Meier analysis of OS and progression-free survival (PFS) demonstrated that the AAT score could significantly stratify patients into low (≤1.8), median (>1.8 to ≤3.0), and high (>3.0) risk groups, with distinct 2-year OS rates of 80.0%, 48.0%, and 4.0%, and PFS rates of 63.3%, 56.0%, and 4.0% in the training cohort, validated in the cohort.

**Conclusions:**

The AAT model effectively stratifies OS and PFS in uHCC patients undergoing TACE plus sintilimab and bevacizumab, guiding personalized treatment decisions.

## Introduction

Hepatocellular carcinoma (HCC) represents a life-threatening malignant tumor with globally prominent rankings in both incidence and mortality rates (1, 2). In China, primary liver cancer ranks as the second leading cause of cancer-related mortality, surpassed only by lung cancer, with HCC accounting for 75–85% of all cases (3, 4). The majority of local patients have a background of HBV infection and consequent cirrhosis, and are typically diagnosed at intermediate or advanced stages, imparting a dismal prognosis with a 5-year survival rate of less than 15% (4, 5). Therefore, finding effective treatment strategies that can prolong survival has become an urgent challenge in the clinical practice of intermediate and advanced-stage HCC.

Although surgical resection remains the primary treatment of choice for HCC, very few patients with intermediate or advanced-stage HCC meet the criteria for surgical intervention (6, 7). A novel strategy of conversion therapy combining local treatment and systemic anti-tumor therapy has achieved breakthrough results in the treatment of unresectable HCC (uHCC) recently, achieving a conversion from the original unresectable state to a resectable state, paving the way for improving the long-term survival rate of advanced HCC patients (7–9). In recent years, multiple studies have reported that the combination of local treatments such as TACE and targeted immunotherapy such as Envafolimab plus Lenvatinib or Sintilimab plus Bevacizumab has effectively improved the conversion rate and prolonged the long-term survival rate of patients in the intermediate or advanced stages (10, 11). However, due to heterogeneity and other reasons, there are still patients who have not reached OR status. Therefore, identifying HCC patients who can truly benefit from the combination therapy of TACE plus Sintilimab and Bevacizumab is an urgent problem to be solved.

This study aimed to facilitate pre-therapeutic efficacy prediction, optimize treatment strategies, and improve long-term survival for patients. We analyzed and identified independent risk factors associated with overall survival in patients with intermediate-advanced HCC undergoing TACE + Sintilimab + Bevacizumab combination therapy, and developed and validated the AAT prognostic scoring model, comprising AFP, ALP, and TBS. The AAT model innovatively achieves stratification of potential HCC patients who responded to TACE + Sintilimab + Bevacizumab combination therapy, which helps optimize individualized treatment decisions and improve patient survival outcomes.

## Materials and methods

### Patients

**Figure 1:**
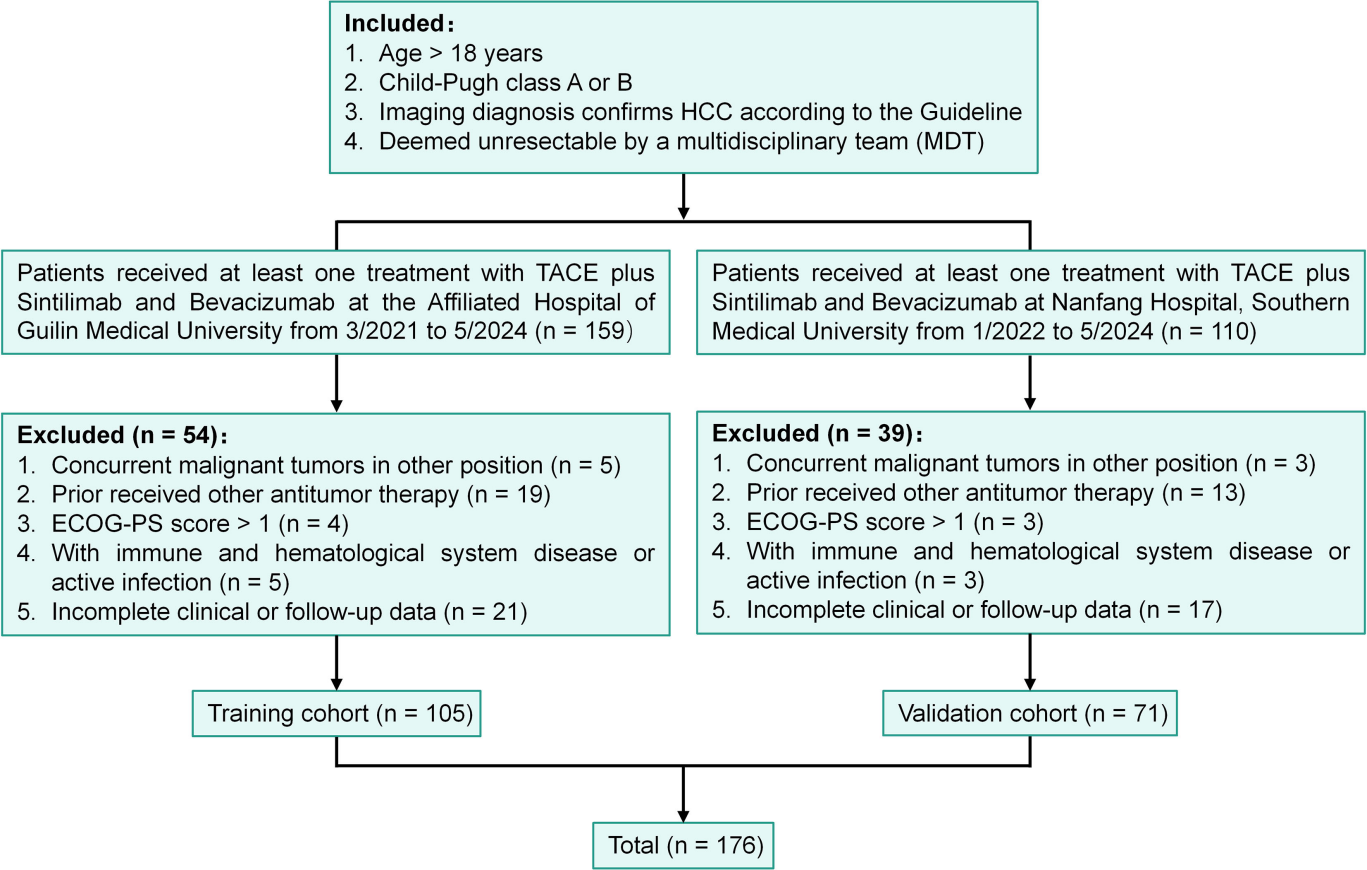
Profile of the study cohorts. This study included 176 unresectable hepatocellular carcinoma (uHCC) patients who received triple therapy (transcatheter arterial chemoembolization [TACE] + Sintilimab + Bevacizumab) from two independent Chinese institutions between March 2021 and May 2024. The training cohort comprised 105 patients from the Affiliated Hospital of Guilin Medical University, and the validation cohort included 71 patients from Nanfang Hospital, Southern Medical University. Key inclusion criteria: age ≥ 18 years, Child-Pugh class A/B, guideline-confirmed uHCC, and unresectable by multidisciplinary team (MDT) evaluation. Exclusion criteria: concurrent other malignancies, prior antitumor therapy, ECOG-PS score > 1, immune/hematological diseases, active infection, or incomplete clinical/follow-up data.

This study retrospectively included a total of 176 uHCC patients who underwent TACE combined with Sintilimab and Bevacizumab triple therapy from two independent institutions in China between March 2021 and May 2024. These patients met the following inclusion criteria: (a) Age ≥ 18 years; (b) Child-Pugh class A or B; (c) Imaging diagnosis confirmed HCC according to guidelines (12); (d) Deemed unresectable by a multidisciplinary team (MDT). And concurrently fulfilled the following exclusion criteria: (a) Concurrent malignant tumors in other positions; (b) Prior received other antitumor therapy; (c) ECOG-PS score > 1; (d) With immune and hematological system disease or active infection; (e) Incomplete clinical or follow-up data. The training cohort in this study consisted of 105 patients from the Affiliated Hospital of Guilin Medical University, and 71 cases from Nanfang Hospital, Southern Medical University were included as the validation cohort (**Figure 1**). This retrospective study was approved by the local Medical Research Ethics Committee, and written informed consent has been obtained from all participants prior to treatment.

### Treatment schedule

All patients in this study underwent one or more TACE procedures, which were performed by experienced interventional radiologists in accordance with established clinical guidelines (12). The TACE operational standards, technical execution, and medication selection were highly consistent and strictly uniform across both participating institutions. Under local anesthesia, femoral artery access was obtained using the Seldinger technique, followed by selective catheterization of the celiac trunk and common hepatic artery for digital subtraction angiography (DSA) to evaluate tumor vascularity and anatomy. A microcatheter was advanced superselectively into tumor-feeding arteries, and an emulsified mixture of chemotherapy drugs (oxaliplatin 75 mg/m^2^) and iodized oil (5–30 mL) was injected, followed by gelatin sponge and polyvinyl alcohol (PVA) particles to achieve complete arterial stasis. TACE was repeated every 4–6 weeks based on clinical assessment, laboratory results, and radiological findings.

All patients received at least one cycle of systemic therapy consisting of sintilimab (200 mg) plus bevacizumab (15 mg/kg) administered intravenously every 21 days. Treatment interruption, dose adjustment, or discontinuation were determined based on the occurrence and severity of treatment-related adverse events (TRAEs).

### Treatment response assessment

Treatment response assessment was performed 4–6 weeks after initial treatment, with tumor response evaluated based on radiological results from CT or MRI scans. According to the modified Response Evaluation Criteria in Solid Tumors (mRECIST) criteria, patients were categorized as follows: complete response (CR), partial response (PR), stable disease (SD), and progressive disease (PD). The objective response rate (ORR) was defined as the proportion of patients achieving either a CR or PR, while the disease control rate (DCR) referred to the percentage of patients with CR, PR, or SD. During the initial treatment and follow-up period, the evaluation of treatment-related adverse events (TRAEs) is used to determine whether to continue treatment or discontinue medication. The criteria for TRAEs are based on the Common Terminology Criteria for Adverse Events (CTCAE) 5.0.

### Follow-up and endpoints

Patients were followed up within a 4- to 8-week interval after the initial treatment, with the follow-up period ending on September 30, 2025. The primary endpoint of this study is overall survival (OS), defined as the time period from the date of initial treatment to the date of death or the last follow-up. The secondary endpoint is progression-free survival (PFS), defined as the time period from the date of the first treatment to recurrence, metastasis, death, or the last follow-up. Data for patients who had not experienced disease progression or were still alive at the last follow-up were processed using the right censoring method.

### Model construction and validation

Univariate Cox regression analysis was performed on variables to identify the risk factors affecting OS in the training cohort, and a risk score was calculated for each factor. Variables with a P-value < 0.05 in the univariate analysis were incorporated into the multivariate Cox regression model to identify independent prognostic factors for OS, with results reported as hazard ratios (HRs) and 95% confidence intervals (CIs). A Cox proportional hazards model was constructed based on the independent prognostic factors, with each patient’s score calculated based on the β coefficients of each factor. The discriminatory ability of the prognostic scoring model was evaluated using the time-dependent receiver operating characteristic (ROC) curve in the training and validation cohorts, and the corresponding area under the curve (AUC) values were computed. The risk model’s stratification performance was assessed in both training and validation cohorts using Kaplan-Meier methods, with survival rate differences estimated by the log-rank test.

### Statistical analysis

Continuous variables were presented as mean ± standard deviation (SD) or median with interquartile range, while categorical variables were expressed as frequencies or percentages. Differences in clinicopathological characteristics were compared using the Student’s *t* test. The predictive accuracy of the model was evaluated by the AUC of the ROC curve. The clinical practicality and calibration performance of the model were evaluated by the decision curve analysis (DCA) and calibration curves. Survival analysis was performed using the Kaplan–Meier method to compare OS and PFS across different risk groups. Hazard ratios (HRs) and 95% confidence intervals (CIs) were calculated. All statistical analyses were conducted using SPSS version 22.0 and R software (version 4.3.1). *p* < 0.05 is considered statistically significant.

## Results

### Patient characteristics

A total of 176 HCC patients who received combined treatment with TACE, Sintilimab, and Bevacizumab were included. Overall, there were 149 men (84.7%) and 27 women (15.3%), with a mean age of 56.63 ± 11.48 years. Some patients have a history of alcohol abuse (n = 55, 31.3%) or smoking (n = 47, 26.7%), while most patients were infected with hepatitis B virus (n = 138, 78.4%). Patients with multiple tumors accounted for 67.6%, with tumor burden score (TBS) of 9.25 ± 3.68. A total of 60.8% of patients had portal vein tumor thrombus (PVTT), and a total of 121 (68.8%) patients had Child-Pugh class A scores, while 48 (27.3%) cases were in BCLC stage B. All had Eastern Cooperative Oncology Group Performance Status (ECOG-PS) of 0 (63.6%) or 1 (36.4%). **Table 1** summarizes the detailed information on the baseline demographic and clinical characteristics of the enrolled patients. There were no significant differences in baseline characteristics between the training cohort and validation cohort (all *p* > 0.05).

**Table 1:** Comparison of clinicopathological characteristics of two groups patients.

Parameter	Total patients (n = 176)	Training cohort (n = 105)	Validation cohort (n = 71)	*P* value
Gender: female/male (n)	27/149	19/86	8/63	0.218
Age (years)	56.63 ± 11.48	56.12 ± 11.99	57.67 ± 10.12	0.264
BMI	21.87 ± 2.70	21.74 ± 2.57	22.05 ± 2.87	0.633
Family history: no/yes (n)	166/10	97/8	69/2	0.320
Alcohol abuse: no/yes (n)	121/55	73/32	48/23	0.788
Smoking: no/yes (n)	129/47	77/28	52/19	0.989
HBsAg: negative/positive (n)	38/138	25/80	13/58	0.384
Tumor size (cm)	9.24 ± 3.93	9.45 ± 3.92	8.94 ± 3.96	0.518
TNb: single/multiple (n)	57/119	39/66	18/53	0.101
TBS	9.25 ± 3.68	9.33 ± 3.72	9.13 ± 3.65	0.227
EHS: no/yes (n)	133/43	80/25	53/18	0.815
PVTT: no/yes (n)	69/107	44/61	25/46	0.372
Child stage: A/B (n)	121/55	68/37	53/18	0.165
BCLC stage: B/C (n)	48/128	30/75	18/53	0.638
ECOG-PS: 0/1	112/64	63/42	49/22	0.223
WBC (×10^9^/L)	6.45 ± 2.46	6.52 ± 2.51	6.34 ± 2.40	0.706
NEUT (×10^9^/L)	4.29 ± 2.26	4.35 ± 2.18	4.20 ± 2.39	0.564
LYMPH (×10^9^/L)	1.33 ± 0.56	1.29 ± 0.50	1.39 ± 0.64	0.272
NLR	3.76 ± 2.65	3.89 ± 2.67	3.53 ± 2.59	0.536
Platelets (×10^9^/L)	197.12 ± 100.71	194.31 ± 102.34	201.28 ± 98.81	0.616
Albumin (g/L)	35.74 ± 6.80	35.35 ± 7.70	36.31 ± 5.21	0.202
Globulin (g/L)	37.54 ± 8.40	38.06 ± 7.74	36.76 ± 9.29	0.399
TBIL (μmol/L, median, IQR)	14.90 (9.42-23.68)	15.60 (9.55-27.06)	14.70 (9.30-20.34)	0.245
DBIL (μmol/L, median, IQR)	6.87 (4.52-13.55)	7.50 (4.50-16.30)	6.20 (4.64-9.90)	0.226
ALBI Score	-2.24 ± 0.67	-2.19 ± 0.75	-2.31 ± 0.52	0.078
ALT (U/L, median, IQR)	32.95 (21.40-50.57)	35.40 (21.0-50.30)	31.60 (22.10-52.10)	0.055
AST (U/L, median, IQR)	50.40 (35.10-96.35)	54.30 (37.85-102.4)	47.60 (33.70-73.30)	0.340
GGT (U/L, median, IQR)	139.50 (88.0-225.0)	137.0 (87.5-234.6)	142.0 (88.0-221.0)	0.884
ALP (U/L)	153.13 ± 79.10	154.29 ± 69.53	151.47 ± 90.08	0.452
AFP (ng/ml, median, IQR)	699.5 (17.42-1210.0)	1044.0 (148.6-1210)	216.0 (7.20-1210.0)	0.630
BUN (mmol/L)	5.41 ± 3.55	5.37 ± 3.67	5.48 ± 3.38	0.795
Cr (μmol/L)	73.17 ± 27.09	74.05 ± 31.23	72.06 ± 19.58	0.076
PT (sec)	13.00 ± 2.05	13.00 ± 1.87	13.01 ± 2.29	0.624
INR	1.13 ± 0.19	1.13 ± 0.17	1.14 ± 0.21	0.339

n, number of patients; BMI, body mass index; HBsAg, hepatitis B surface antigen; TNb, tumor number; EHS, extrahepatic spread; PVTT, portal vein tumor thrombus; BCLC, Barcelona Clinic Liver Cancer; TBS, tumor burden score; ECOG-PS, eastern cooperative oncology group performance status; WBC, white blood cell; NEUT, neutrophil count; LYMPH, lymphocyte count; NLR, neutrophil to lymphocyte ratio; TBIL, total bilirubin; IQR, interquartile range; DBIL, direct bilirubin; ALT, alanine aminotransferase; AST, aspartate aminotransferase; GGT, Gamma-glutamyl transpeptidase; ALP, alkaline phosphatase; AFP, alpha-fetoprotein; ALBI, albumin-bilirubin; BUN, blood urea nitrogen; Cr, creatinine; PT, prothrombin time; INR, international normalized ratio.

### Independent factors associated with OS

Univariate analysis identified five indicators that were significantly correlated with OS in HCC patients receiving TACE, Sintilimab, and Bevacizumab combination therapy: AFP > 400 ng/mL (*p* = 0.004), ALP > 120 U/L (*p* < 0.001), TBS > 8 (*p* = 0.049), presence of PVTT (*p* = 0.033), and neutrophil-to-lymphocyte ratio (NLR) > 2.3 (*p* = 0.043). The details are presented in **Table 2**. Furthermore, **Table 3** presents the results of the multivariate analysis, in which PVTT (present *vs.* absent, *p* = 0.088) and NLR (> 2.3 *vs.* ≤ 2.3, *p* = 0.581) are not significant, whereas AFP > 400 ng/mL (*p* = 0.003), ALP > 120 U/L (*p* < 0.001), and TBS > 8 (*p* = 0.005) are significant independent risk factors for OS. Their β-estimate (95% CI) were 1.024 (0.356–1.693), 1.404 (0.738–2.069), 0.881 (0.267–1.495), respectively.

**Table 2:** Univariate Cox regression analyses of OS in the training cohort.

Variable	HR	95% CI	*P* value
Gender (male *vs.* female)	0.838	0.423-1.662	0.613
Age, years (> 65 *vs.* ≤ 65)	0.916	0.484-1.735	0.788
BMI (> 22 *vs.* ≤ 22)	0.594	0.326-1.082	0.089
Alcohol abuse (present *vs*. absent)	0.717	0.397-1.295	0.270
HBsAg (positive *vs*. negative)	1.336	0.691-2.583	0.390
Tumor size, cm (> 5 *vs*. ≤ 5)	1.392	0.630-3.073	0.414
Tumor number (multiple *vs*. single)	0.927	0.539-1.592	0.783
TBS (> 8 *vs*. ≤ 8)	1.813	1.002-3.279	**0.049**
EHS (present *vs*. absent)	1.694	0.965-2.972	0.066
PVTT (present *vs*. absent)	1.843	1.049-3.237	**0.033**
ECOG-PS (1 *vs*. 0)	0.822	0.476-1.421	0.484
NLR (> 2.3 *vs*. ≤ 2.3)	1.935	1.021-3.669	**0.043**
ALP, U/L (> 120 *vs*. ≤ 120)	3.585	1.888-6.807	**< 0.001**
AFP, ng/ml (> 400 *vs*. ≤ 400)	2.664	1.376-5.159	**0.004**

HR, hazard ratio; CI, confidence interval; BMI, body mass index; HBsAg, hepatitis B surface antigen; TBS, tumor burden score; EHS, extrahepatic spread; PVTT, portal vein tumor thrombus; ECOG-PS, eastern cooperative oncology group performance status; NLR, neutrophil to lymphocyte ratio; ALP, alkaline phosphatase; AFP, alpha-fetoprotein.

The bold values indicate statistically significant prognostic factors with P < 0.05.

**Table 3:** Multivariable Cox regression analyses of OS in the training cohort.

Variable	β-estimate	HR (95% CI)	*P* value
AFP, ng/ml (> 400 *vs*. ≤ 400)	1.024	2.785 (1.427-5.435)	**0.003**
ALP, U/L (> 120 *vs*. ≤ 120)	1.404	4.070 (2.092-7.920)	**0.000**
TBS (> 8 *vs*. ≤ 8)	0.881	2.413 (1.306-4.458)	**0.005**
PVTT (present *vs*. absent)		1.656 (0.928-2.955)	0.088
NLR (> 2.3 *vs*. ≤ 2.3)		1.210 (0.615-2.379)	0.581

HR, hazard ratio; CI, confidence interval; ALP, alkaline phosphatase; AFP, alpha-fetoprotein; TBS, tumor burden score; PVTT, portal vein tumor thrombus; NLR, neutrophil to lymphocyte ratio.

The bold values indicate statistically significant independent prognostic factors with P < 0.05.

### Development of AAT model

The novel prognostic AAT model for TACE plus Sintilimab and Bevacizumab treated HCC patients is constructed based on the above three independent prognostic factors (AFP, ALP, TBS). The score for each factor is generated according to their β score, and the prognostic evaluation model is the sum of the scores for each factor. The hazard function formula for AAT model was derived as follows: AAT score = 1.024*AFP + 1.404*ALP + 0.881*TBS (χ^2^ = 42.077, *p* < 0.001). Assign values to each of the three independent predictors for each patient, such as assigning 0 points if AFP ≤ 400 and 1 point if AFP > 400 ng/mL, 0 points if ALP ≤ 120 U/L and 1 point if ALP > 120 U/L, 0 points if TBS ≤ 8 and 1 point if TBS > 8, and calculate the AAT scores for all patients.

### Performance and validation of AAT model

**Figure 2:**
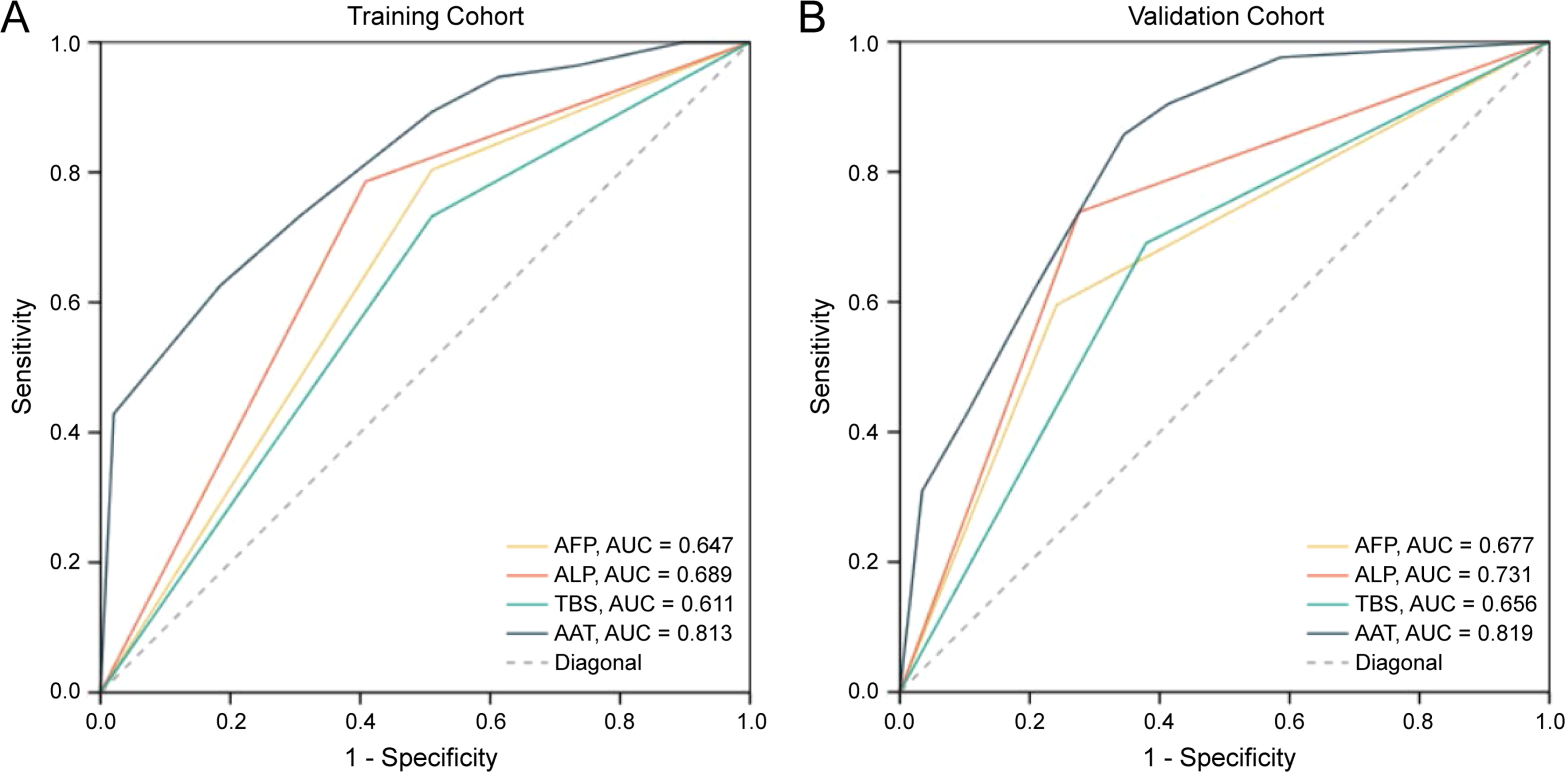
Comparison of receiver operating characteristic (ROC) curves for predicting overall survival between AAT model and other individual indicators in training cohort **(A)** and validation cohort **(B)**. **(A)** Training cohort: AAT model AUC = 0.813 (95% CI: 0.733–0.892), *vs*. AFP (0.647, 95% CI: 0.540–0.754), ALP (0.689, 95% CI: 0.585–0.792), TBS (0.611, 95% CI: 0.502–0.720). **(B)** Validation cohort: AAT model AUC = 0.819 (95% CI: 0.717–0.920), *vs.* AFP (0.677, 95% CI: 0.550–0.804), ALP (0.731, 95% CI: 0.609–0.853), TBS (0.656, 95% CI: 0.524–0.787).

**Figure 3:**
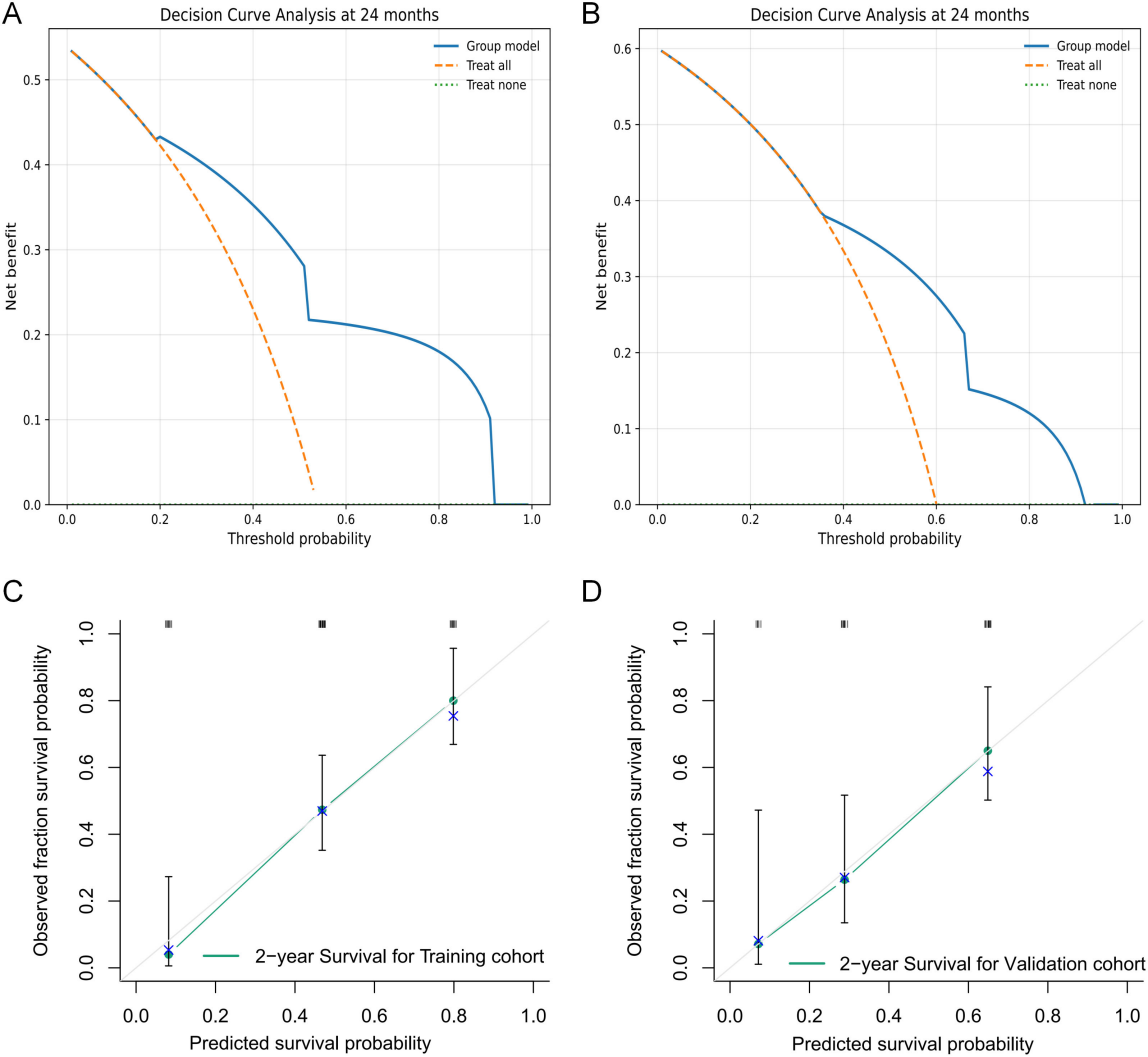
DCA and calibration curves of the AAT score. DCA in training **(A)** and validation **(B)** cohorts, comparing net benefit of AAT with treat-all/treat-none strategies. Calibration curves in training **(C)** and validation **(D)** cohorts.

To evaluate the discriminative ability of the AAT model for OS of patients, we plotted the receiver operating characteristic curve of the subjects and calculated the AUC. Within the training cohort, the AAT model demonstrated superior predictive value compared to single variables AFP, ALP, and TBS, with an AUC of 0.813 (95% CI = 0.733–0.892) (**Figure 2A**). And the AUCs for the other three indicators were 0.647 (95% CI = 0.540–0.754), 0.689 (95% CI = 0.585–0.792), and 0.611 (95% CI = 0.502–0.720), respectively. In the validation group, the AAT model showed equally excellent predictive value with an AUC of 0.819 (95% CI = 0.717–0.920) (**Figure 2B**), while the AUC of the other three indicators were 0.677 (95% CI = 0.550–0.804), 0.731 (95% CI = 0.609–0.853), and 0.656 (95% CI = 0.524–0.787), respectively. In addition, we further evaluated the clinical utility and calibration performance of AAT scoring. As shown in **Figures 3A, B**, decision curve analysis (DCA) revealed that the AAT score yielded superior net clinical benefits over both the “treat-all” and “treat-none” strategies across a broad range of threshold probabilities in both the training and validation cohorts. Meanwhile, the calibration curves confirmed excellent agreement between the predicted and observed 2-year survival probabilities in both cohorts (**Figures 3C, D**).

### Efficacy and response evaluation

The median OS of the training cohort was 17.0 months (interquartile range [IQR], 9.0–29.0 months) (**Supplementary Figure 1A**). The 1-, 2-, and 3-year survival rates were 63.8%, 46.3%, and 46.3%, respectively. The median OS of the validation group patients was 18.0 months (IQR, 12.0–30.0 months), with 1-, 2-, and 3-year survival rates of 78.9%, 41.8%, and 40.2%, respectively (**Supplementary Figure 1B**). Meanwhile, the median PFS for the training and validation cohort were 11.0 months (IQR, 6.0–22.0 months) and 12.0 months (IQR, 8.0–24.0 months), respectively. The 1-, 2-, and 3-year PFS rates of these two cohorts were 50.5%, 33.9%, and 33.9%; and 56.3%, 34.7%, and 34.7% (**Supplementary Figure 2**).

According to the mRECIST criteria, patients’ response to TACE plus Sintilimab and Bevacizumab combination treatment was measured based on the four different response guidelines. Patients who achieve complete response (CR) or partial response (PR) are considered to have objective response, while those who achieve CR or PR and stable disease (SD) are defined as disease control. The efficacy response evaluations of all patients, as well as the training and validation groups, are summarized in **Supplementary Table 1**. Among a total of 176 patients, the number of cases with CR, PR, SD and progressive disease (PD) were 44 (25.00%), 58 (32.95%), 37 (21.02%), 37 (21.02%), with objective response rate (ORR) and disease control rate (DCR) of 57.95%, 78.97%, respectively. The ORR and DCR of the training queue and validation queue are similar among all patients, at 57.14%, 78.09%, and 59.15%, 80.28%, respectively.

### Prediction performance of AAT model for OS/PFS

**Figure 4:**
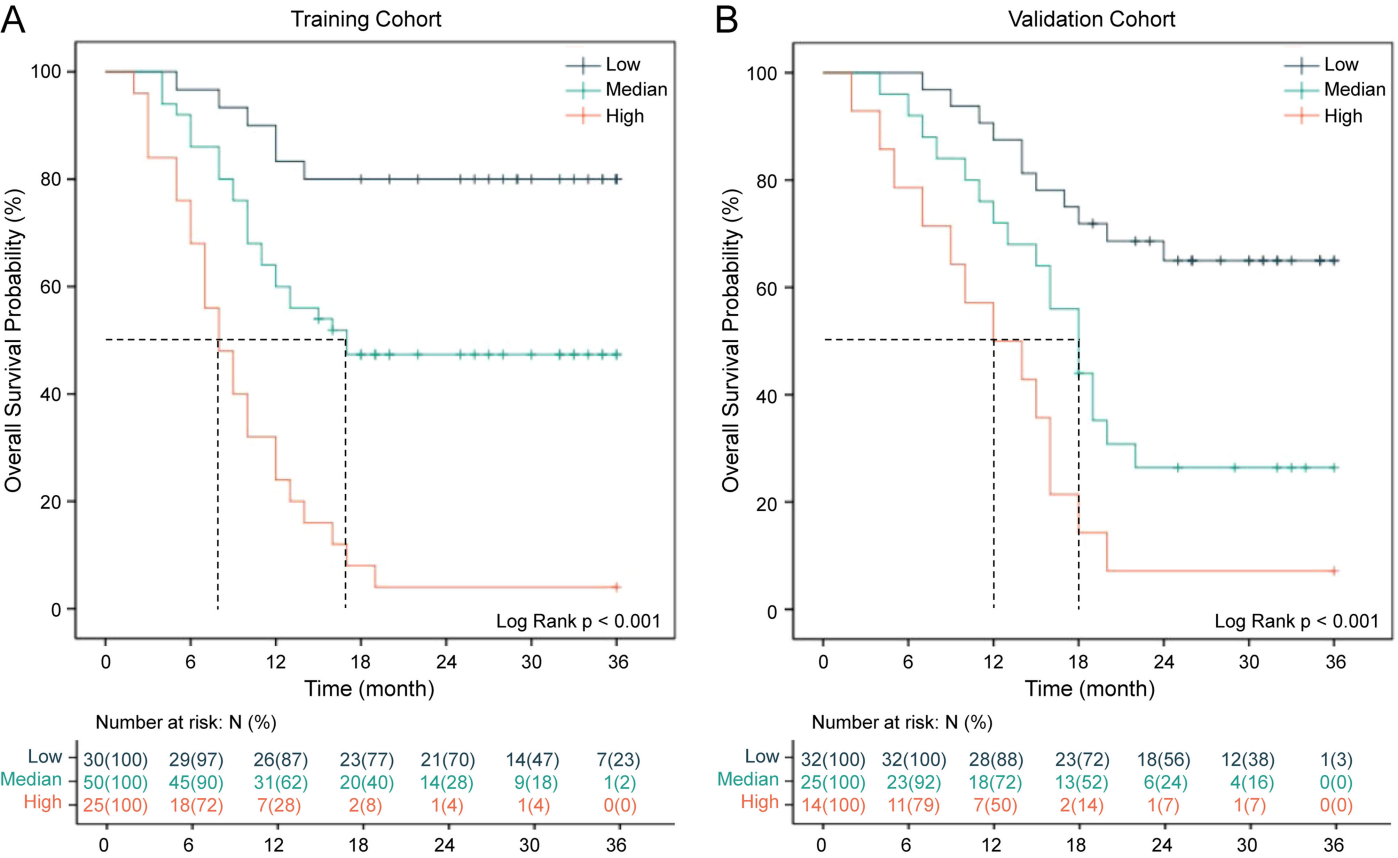
Kaplan-Meier curve of overall survival (OS) for each group of patients stratified by AAT scoring model in the training **(A)** and validation **(B)** cohort. **(A)** Training cohort: 2-year OS rates = 80.0% (Low) *vs*. 48.0% (Median) *vs*. 4.0% (High) (*p* < 0.001). **(B)** Validation cohort: 2-year OS rates = 68.6% (Low) *vs*. 26.4% (Median) *vs*. 7.1% (High) (*p* < 0.001).

**Figure 5:**
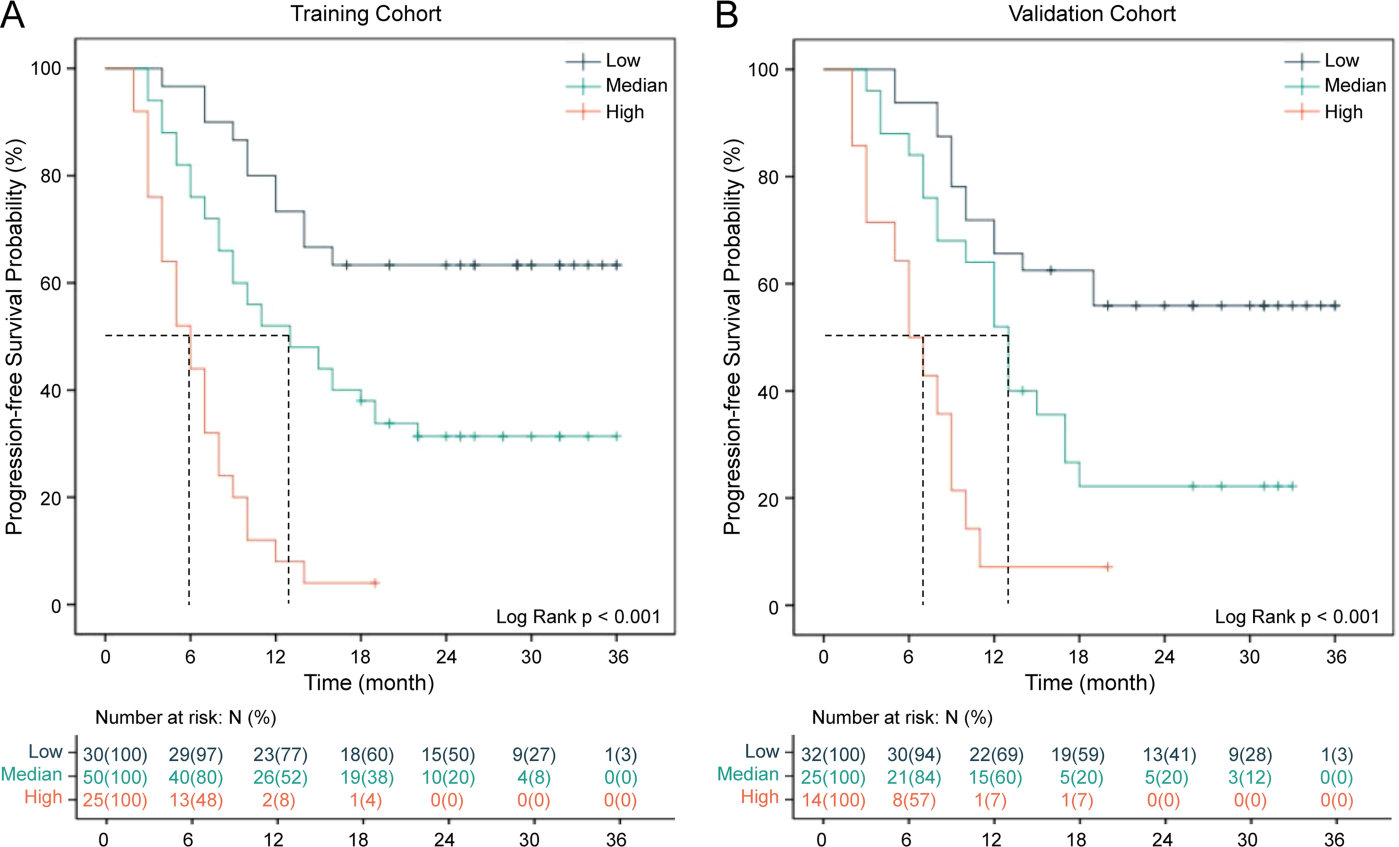
Kaplan-Meier curve of progression-free survival (PFS) for each group of patients stratified by AAT scoring model in the training **(A)** and validation **(B)** cohort. **(A)** Training cohort: 2-year PFS rates = 63.3% (Low) *vs*. 56.0% (Median) *vs*. 4.0% (High) (*p* < 0.001). **(B)** Validation cohort: 2-year PFS rates = 55.9% (Low) *vs*. 22.2% (Median) *vs*. 7.1% (High) (*p* < 0.001).

Patients were categorized into three groups based on their AAT score reflecting their prognosis risk: Low group (≤ 1.8), Median group (>1.8 to ≤ 3.0), and High group (> 3.0). The 2-year OS rates of the three groups were 80.0%, 48.0%, and 4.0% in training cohort, respectively (*p* < 0.001, **Figure 4A**). The 2-year OS rates for the Low/Median/High groups in validation cohort are as follows: 68.6%, 26.4%, 7.1% (*p* < 0.001, **Figure 4B**). The AAT model also shows significant stratification ability in predicting PFS, the 2-year PFS rates in training cohort for the Low/Median/High groups were 63.3%, 56.0%, and 4.0%, respectively (*p* < 0.001, **Figure 5A**); whereas in the validation cohort, these rates were 55.9%, 22.2%, and 7.1% (*p* < 0.001, **Figure 5B**).

## Discussion

The combination of local treatment and systemic anti-tumor therapy is increasingly being used in patients with intermediate-advanced HCC (13, 14). However, long-term survival remains limited due to primary drug insensitivity and the risk of acquired resistance (15–17). Pre-therapeutic prediction of OS to identify high-risk patients could guide personalized treatment decisions. We therefore developed and validated the AAT model (comprising AFP, ALP, and TBS) for HCC patients undergoing TACE + Sintilimab + Bevacizumab, which significantly predicted both OS and PFS in patients. Using the AAT model to score patients’ risk can effectively identify high-risk populations, guide personalized treatment decisions, and improve patient survival rates.

Although surgical resection is an important approach for achieving long-term survival in patients with hepatocellular carcinoma, those with unresectable HCC lack this curative option. Mounting evidence suggests that both local therapy and systemic anti-tumor therapy bring new hope to uHCC patients by increasing resection rates, decreasing postoperative recurrence and metastasis, and paving the way for improved prognosis (7, 18–20). In recent years, multiple phase II and III trials have confirmed that the combination of antiangiogenic agents and immune checkpoint inhibitors in systemic anti-tumor therapy significantly improves survival outcomes and increases the surgical conversion rate in patients with advanced HCC (20–24). For example, the phase III study of CARES-310 found that the combination of anti-PD-1 antibody Camrelizumab plus the VEGFR2-targeted tyrosine-kinase inhibitor (TKI) Rivoceranib is more effective than sorafenib in first-line treatment of uHCC (24). Additionally, other studies have revealed that the combination of local treatments such as TACE, hepatic arterial infusion chemotherapy (HAIC), and systemic anti-tumor therapy can further improve the conversion rate and prolong prognosis (11, 25). Zhang et al. reported in a phase II study that the triple regimen of Camrelizumab, Lenvatinib, and RALOX-HAIC has shown significant anti-tumor efficacy in the treatment of patients with intermediate-to-advanced HCC (25). Similarly, a phase II study by Mao et al. (NCT04796025) demonstrated that TACE combined with Sintilimab and Bevacizumab achieved an objective response rate (ORR) of 70.6% in advanced HCC, outperforming the Sintilimab and Bevacizumab biosimilar regimen from the ORIENT-32 trial (11). Further supporting this approach, Qin et al. confirmed the efficacy and manageable safety of TACE plus Sintilimab and Bevacizumab biosimilar in uHCC, with an ORR of 59.2% (26). Based on these findings, this study recruited HCC patients from two institutions who received TACE combined with Sintilimab and Bevacizumab treatment, and observed an ORR of 57.95%, which is consistent with previous reports. In terms of survival outcomes, the mean OS was 21.7 months in the training cohort and 22.5 months in the validation cohort, while the mean PFS was 17.9 months and 18.6 months, respectively. Building on the established survival benefit of the systemic anti-PD-1 and antiangiogenic combination (Sintilimab plus Bevacizumab) in intermediate-advanced HCC, this study incorporated TACE as a local therapeutic modality. Given the established role of TACE in converting unresectable HCC (18, 27), our clinical data further substantiate the synergistic antitumor effect of TACE combined with immunotherapy and targeted therapy in intermediate-advanced HCC. Furthermore, the incidence of treatment-related adverse events (TRAEs) in our cohort was consistent with previous reports: any-grade and grade ≥3 TRAEs occurred in 93.18% and 22.16% of patients, respectively (**Supplementary Table 2**). These findings support the favorable safety profile of this combination regimen, complementing its promising efficacy.

Our study identified three independent risk factors significantly associated with overall survival in HCC patients treated with TACE + Sintilimab + Bevacizumab combination therapy through Cox Proportional-Hazards Model: AFP, ALP, and TBS. Based on these factors, we constructed an AAT score to predict the potential population that is effective for this treatment regimen. AFP serves as a crucial biomarker for early diagnosis of HCC, holds significant value in predicting response to immunotherapy and/or targeted therapy (28, 29), and also demonstrates utility as a prognostic indicator in HCC patients undergoing TACE (30, 31). Elevated levels of ALP in HCC patients often indicate tumor-associated inflammatory responses, and even disease progression. Research has shown that ALP > 120 U/L is associated with poor prognosis in HCC patients (32). Elevated ALP has been shown to be an independent prognostic factor for OS in hepatocellular carcinoma patients treated with TACE or in combination with targeted therapy agents such as Lenvatinib and Axitinib (30, 33, 34). Both tumor size and number are critical factors influencing the treatment decision in HCC, and larger tumors and more lesions are associated with poorer prognosis (35–37). Patients with higher tumor burden are more likely to experience deterioration of liver function reserve after TACE treatment (31, 38). Henrique et al. found that TBS > 8.0 is associated with OS in intermediate-stage HCC (39). In a phase 3 trial of Lenvatinib plus PD-1 inhibitors for the treatment of uHCC, TBS less than 8 was independently associated with a significantly longer OS (34). Based on the above three factors, we constructed an AAT model that can effectively stratify the potential responders to TACE + Sintilimab + Bevacizumab combination therapy. The AUC of the ROC curve in the training cohort and validation cohort of the AAT model is 0.813 and 0.819, respectively. In our cohort, the AUC values of ALBI (40), HAP (41), and our novel AAT score were 0.735, 0.757, and 0.813 in the training set, whereas the corresponding values in the validation set were 0.679, 0.722, and 0.819. Compared with these two conventional scoring systems, the AAT score displayed stronger predictive discriminative ability. Overall, our newly developed model yielded better prognostic performance than traditional liver function and prognostic scores in patients receiving triple therapy. Notably, the AAT score was derived from routine pretreatment indicators, rendering it concise and clinically feasible for individualized treatment decision-making. Furthermore, DCA revealed that the AAT score yielded superior net clinical benefits over both the “treat-all” and “treat-none” strategies across a broad range of threshold probabilities in both the training and validation cohorts. Meanwhile, the calibration curves confirmed excellent agreement between the predicted and observed 2-year survival probabilities in both cohorts. Collectively, these findings indicate that the AAT score is not only statistically robust but also clinically practical for guiding individualized treatment decisions in uHCC patients receiving triple therapy.

Patients in the training cohort were stratified by the AAT score into Low group (≤ 1.8), Median group (>1.8 to ≤ 3.0), and High group (> 3.0), with corresponding 2-year survival rates of 80.0%, 48.0%, and 4.0%, and similar data were also confirmed in the validation cohort (68.6%, 26.4%, and 7.1%). Meanwhile, the AAT model can also be used to predict PFS.

The AAT score, derived from routine non-invasive pretreatment indicators, enables precise risk stratification, identifies potential non-responders to TACE plus sintilimab and bevacizumab, and guides individualized treatment adjustments to avoid ineffective interventions and improve patient outcomes. Nevertheless, several limitations of this study should be acknowledged. First, the retrospective design inherently carries potential selection bias. Second, generalizability is limited by the modest sample size (n = 176) and the homogeneous cohort from two southern Chinese tertiary hepatobiliary centers, consisting predominantly of Asian patients with HBV-related HCC. The model’s performance in non-Asian populations, other etiologies (e.g., HCV, alcohol-related HCC), and diverse healthcare settings, as well as its clinical utility, require validation in prospective clinical trials. Third, inter-individual heterogeneity in treatment intensity (TACE cycles and systemic therapy infusions) reflects real-world customization based on tumor response, liver function, and patient tolerance—not a study flaw—but introduces statistical complexity. Owing to limited subgroup sample sizes and the risk of immortal time bias from post-treatment dynamic variables, we did not include TACE cycles as a baseline covariate or perform stratified analyses. Furthermore, as the AAT score is designed strictly as a pretreatment decision-making tool, detailed cross-stratum analysis of post-treatment events (including specific toxicities, dose adjustments, and conversion to resectability) was limited by the modest sample size, which may introduce residual confounding. Future large-scale prospective studies are required to continuously monitor these longitudinal post-baseline variables. Future large prospective studies using landmark analysis or marginal structural models should explore treatment intensity’s modifying effect on the AAT score’s prognostic value. Finally, it remains undetermined whether the AAT score specifically predicts the therapeutic benefit of this triple therapy or merely identifies patients with an inherently poor prognosis in unresectable HCC. Its applicability to patients receiving alternative treatment regimens warrants further investigation.

Despite these limitations, the AAT score still provides a practical, guideline-aligned clinical decision-making framework for individualized management of advanced HCC patients receiving TACE plus immunotherapy. Based on the 2022 Chinese Guidelines for the Diagnosis and Treatment of Primary Liver Cancer and our risk stratification results, we propose the following tiered management recommendations: 1) For low-risk patients (≤ 1.8): Standard TACE combined with sintilimab and bevacizumab therapy is recommended, with regular imaging and laboratory follow-up every 8 weeks, consistent with the guideline-recommended assessment interval for patients with stable disease. 2) For intermediate-risk patients (>1.8 to ≤ 3.0): Given their higher risk of early progression, consideration may be given to intensifying treatment (e.g., adjusting TACE frequency based on mRECIST response, or optimizing systemic therapy) and shortening follow-up to every 4–6 weeks, in line with the guideline’s “on-demand” TACE principle and individualized risk-based monitoring. 3) For high-risk patients (> 3.0): These patients are at significant risk of poor response to standard therapy; early enrollment in clinical trials or timely switching to alternative treatment regimens is strongly recommended, as guided by the guidelines for managing patients with high progression risk.

In conclusion, this study developed and validated the AAT score, a novel non-invasive pretreatment prognostic tool for HBV-related unresectable HCC patients undergoing TACE combined with sintilimab and bevacizumab. This scoring system enables precise pretreatment risk stratification and provides clinicians with a robust framework for individualized decision-making. These findings support the AAT score’s potential to improve patient outcomes, though its generalizability and clinical utility require validation in future prospective studies.

## Data Availability

The raw data supporting the conclusions of this article will be made available by the authors, without undue reservation.
