# Supplementary figures and images for: AAT score based on pretreatment indicators predicts outcomes in unresectable HCC patients treated with TACE, Sintilimab, and Bevacizumab
Source: Front Oncol. 2026 Jun 10;16:1867932. doi: 10.3389/fonc.2026.1867932 (PMC13290531; doi:10.3389/fonc.2026.1867932)

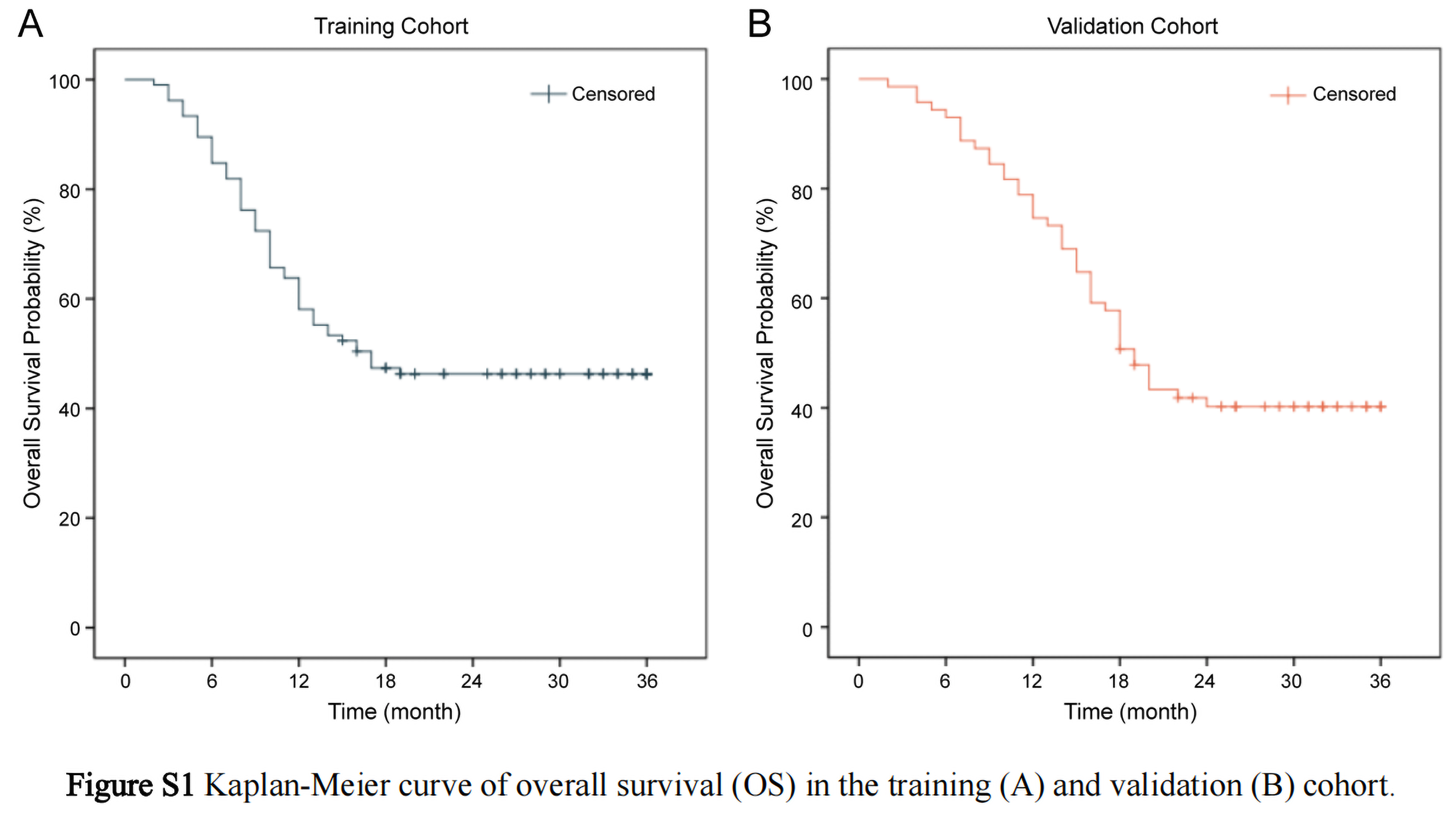

Supplement: Supplementary file 1 [file Image1.tif]

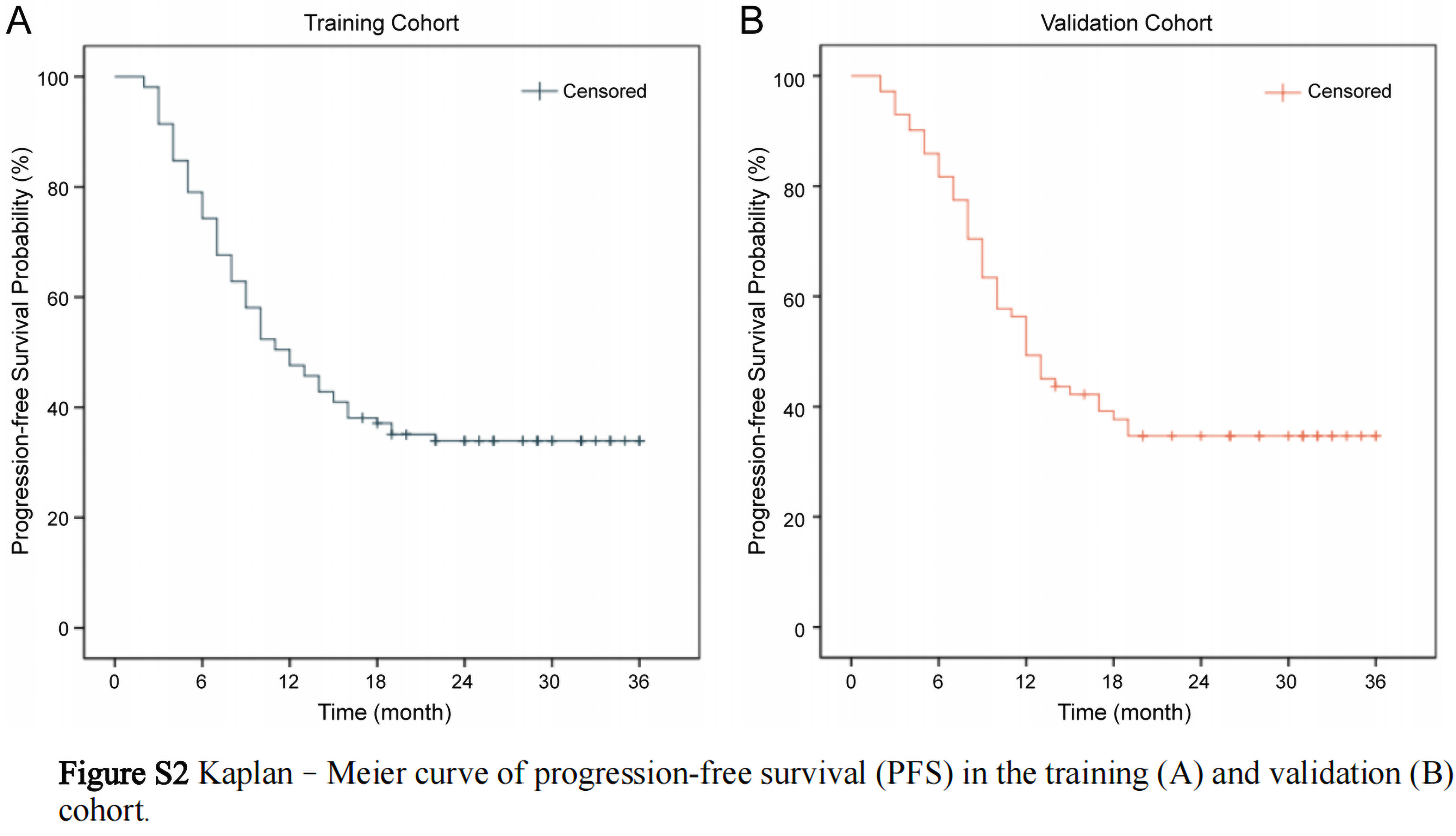

Supplement: Supplementary file 2 [file Image2.tif]

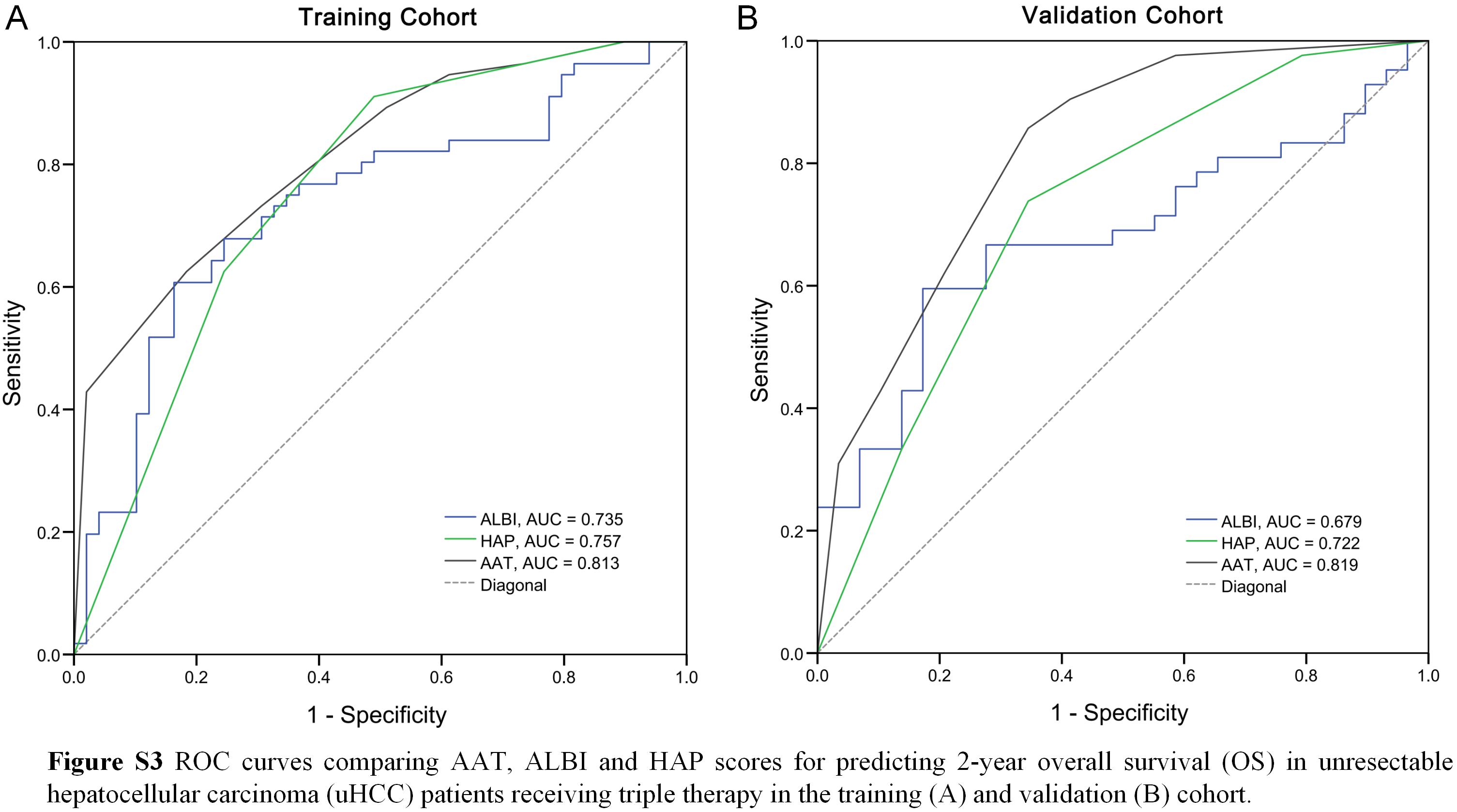

Supplement: Supplementary file 3 [file Image3.tif]
