# Supplementary material for: AAT score based on pretreatment indicators predicts outcomes in unresectable HCC patients treated with TACE, Sintilimab, and Bevacizumab
Source: Front Oncol. 2026 Jun 10;16:1867932. doi: 10.3389/fonc.2026.1867932 (PMC13290531; doi:10.3389/fonc.2026.1867932)
Supplement: Supplementary file 4 [file Table1.doc]

**Table S1. Response according to mRECIST in the training and validation cohort.**

| Variable | Total patients  (n = 176, %) | Training cohort  (n = 105, %) | Validation cohort  (n = 71, %) |
| --- | --- | --- | --- |
| Complete response | 44 (25.00) | 26 (24.76) | 18 (25.35) |
| Partial response | 58 (32.95) | 34 (32.38) | 24 (33.80) |
| Stable disease | 37 (21.02) | 22 (20.95) | 15 (21.12) |
| Progressive disease | 37 (21.02) | 23 (21.90) | 14 (19.71) |
| Objective response rate | 102 (57.95) | 60 (57.14) | 42 (59.15) |
| Disease control rate | 139 (78.97) | 82 (78.09) | 57 (80.28) |

**Abbreviations:** n, number of patients; mRECIST modified response evaluation criteria in solid tumors.
