# Supplementary material for: AAT score based on pretreatment indicators predicts outcomes in unresectable HCC patients treated with TACE, Sintilimab, and Bevacizumab
Source: Front Oncol. 2026 Jun 10;16:1867932. doi: 10.3389/fonc.2026.1867932 (PMC13290531; doi:10.3389/fonc.2026.1867932)
Supplement: Supplementary file 5 [file Table2.docx]

**Table S2** Treatment-Related Adverse Events (TRAEs) Observed in the Study Cohort.

| Adverse events | Any grade, n (%) | Grade ≥3, n (%) |
| --- | --- | --- |
| Hypertension | 63 (35.80) | 21 (11.93) |
| Decreased appetite | 51 (28.98) | 0 (0.00) |
| Fatigue | 46 (26.14) | 4 (2.27) |
| Abdominal pain | 44 (25.00) | 2 (1.14) |
| Nausea | 42 (23.86) | 0 (0.00) |
| Elevated ALT/AST | 41 (23.30) | 0 (0.00) |
| Diarrhea | 38 (21.59) | 5 (2.84) |
| Proteinuria | 37 (21.02) | 6 (3.41) |
| Hand-foot syndrome | 35 (19.89) | 2 (1.14) |
| Pyrexia | 34 (19.32) | 0 (0.00) |
| Hematologic toxicity (WBC/neutropenia) | 31 (17.61) | 2 (1.14) |
| Rash/Pruritus | 30 (17.05) | 0 (0.00) |
| Decreased platelet count | 28 (15.91) | 3 (1.70) |
| Hypoalbuminemia | 25 (14.20) | 5 (2.84) |
| Anemia | 21 (11.93) | 7 (3.98) |
| Bleeding events | 10 (5.68) | 2 (1.14) |
| Total patients with ≥1 TRAE | 164 (93.18) | 39 (22.16) |

**Abbreviations:** TRAEs, Treatment-Related Adverse Events; n, number of patients; ALT, alanine aminotransferase; AST, aspartate aminotransferase; WBC, white blood cell count
